# Supplementary material for: Protocol Article: A Cross-Sectional Evaluation of Children’s Feet and Lower Extremities
Source: Methods Protoc. 2023 Dec 1;6(6):115. doi: 10.3390/mps6060115 (PMC10745981; doi:10.3390/mps6060115)
Supplement: Supplementary file 1 [file mps-06-00115-s001.zip › mps-2678253-supplementary materials.pdf]

DATA fra Fodprojekt

Skole:	
Klasse:	
Navn:	
Patient ID:	
Undersøgelsesdato:	
Undersøger:	

Starts position = 0° for goniometer

Start ved vinkel

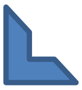

Start ved udstrakt

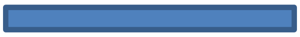

start ved samlet

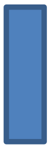

PATIENT ID	

Inspektion Forfra

Fodlængde, cm	Højre:___________<br>Venstre:_________	Måles fra hælen til længste tå. Måleinstrument
Fodbredde, cm	Højre:___________<br>Venstre:_________	Måles på bredeste sted<br>Måleinstrument
Svanghøjde	Højre:____________<br>Venstre:__________	Menes der navicular height<br>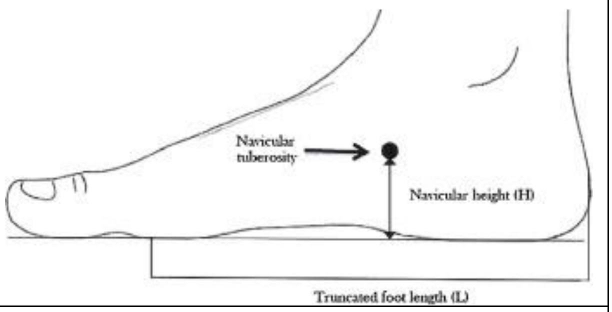
Feiisline	Højre:<br>☐ Over feisslinje<br>☐ På feisslinje<br>☐ Under feisslinje<br>Venstre:<br>☐ Over feisslinje<br>☐ På feisslinje<br>☐ Under feisslinje	Er Navicular:<br>• Over feisslinje<br>• På feisslinje<br>• Under feisslinje
Hallux valgus	Højre:___________<br>Venstre:_________	Måles med lille goniometer<br><br>**Normal: Valgus 0-10°**
Knæ valgus/varus (grader)	Højre:___________<br>Venstre:_________	Vinkel mellem femur og tibia. Valgus som positiv vinkel, varus som negativ.<br><br>**Normal: Valgus 10°**
Nedgroede negle	☐ Ingen nedgroede negle<br><br>**Højre**<br>☐ Medialt 1. tå<br>☐ Lateralt 1. tå<br>☐ Øvrige tæer<br>**Venstre**<br>☐ Medialt 1. tå<br>☐ Lateralt 1. tå<br>☐ Øvrige tæer	

PATIENT ID	

Inspektion Bagfra

Hælvalgus	Højre:___________<br><br>Venstre:_________	Vinkel mellem calcaneus og tibia<br>Valgus som positiv vinkel, varus som negativ.<br><br>Normal: 0-5°
Tåstand	Højre	☐ Hæl i varus<br>☐ Hæl i vaLgus
	Venstre	☐ Hæl i varus<br>☐ Hæl i VaLgus

Inspektion Liggende på Ryggen

Hoftebevægelighed	Højre	Fleksion: (0-140) _________<br>Indadrotation: (0-60) _________<br>Udadrotation: (0-60) _________<br><br>Anterversion: ___________<br><br>Udadføring (abduktion): (0-60) ________<br>Indadføring (adduktion): (0-45) ________
	Venstre	Fleksion: (0-140) _________<br>Indadrotation: (0-60) _________<br>Udadrotation: (0-60) _________<br><br>Anterversion: ___________<br><br>Udadføring (abduktion): (0-60) ________<br>Indadføring (adduktion): (0-45) ________
Knæbevægelighed	Højre	Fleksion: (140) ___________<br>Ekstension: (0 - -10) ___________
	Venstre	Fleksion: (140) ___________<br>Ekstension: (0 - -10) ___________
Ankelled	Højre	Dorsifleksion bøjet knæ: (20°) ________<br>Dorsifleksion strakt knæ: (20°) _______<br>Plantarfleksion: (45°) _________
	Venstre	Dorsifleksion bøjet knæ: (20°) ________<br>Dorsifleksion strakt knæ: (20°) _______<br>Plantarfleksion: (45°) _________

PATIENT ID	

Bagfodsbevægelighed<br><br>Normal 20° ind- og udad	Højre	☐ Øget bevægelse<br>☐ Normal<br>☐ Nedsat bevægelse<br>☐ Ingen bevægelse
	Venstre	☐ Øget bevægelse<br>☐ Normal<br>☐ Nedsat bevægelse<br>☐ Ingen bevægelse
Forfodsbevægelighed<br><br>Supination 30°	Højre	☐ Øget bevægelse<br>☐ Normal<br>☐ Nedsat bevægelse<br>☐ Ingen bevægelse
	Venstre	☐ Øget bevægelse<br>☐ Normal<br>☐ Nedsat bevægelse<br>☐ Ingen bevægelse
Forfodsbevægelighed<br><br>Pronation 30°	Højre	☐ Øget bevægelse<br>☐ Normal<br>☐ Nedsat bevægelse<br>☐ Ingen bevægelse
	Venstre	☐ Øget bevægelse<br>☐ Normal<br>☐ Nedsat bevægelse<br>☐ Ingen bevægelse
Callositeter<br><br>Indtegnes		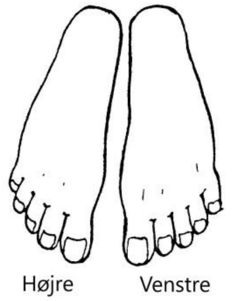

PATIENT ID	

Inspektion Bugleje

Tibiatorsionsvinkel	Højre:_________<br>Venstre:_______	Vinkel mellem intermalleolærlinje og femur
Foot-thigh vinkel	Højre:_________<br>Venstre:_______	Vinkel mellem foden i neutralstilling og femur
Metatarsus varus<br><br>Normal lige inderside	Højre:	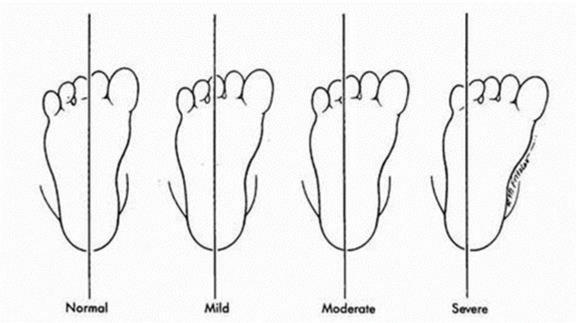<br>☐ Ingen<br>☐ Mild<br>☐ Moderat<br>☐ Svær
	Venstre	☐ Ingen<br>☐ Mild<br>☐ Moderat<br>☐ Svær
Fodvorter<br>(Indtegnes)		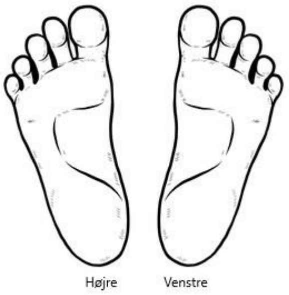
Callositeter		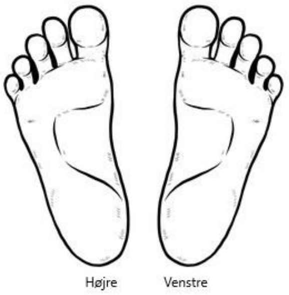

PATIENT ID	

Generelle mål

Højde, cm	
Vægt, kg	
Aktuelle smerter<br><br>Marker på figuren	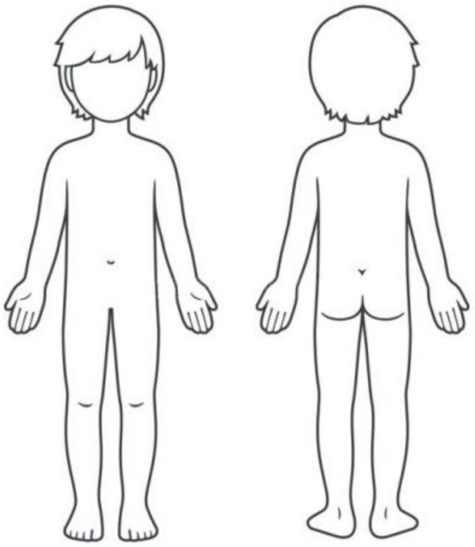
NRS-placering	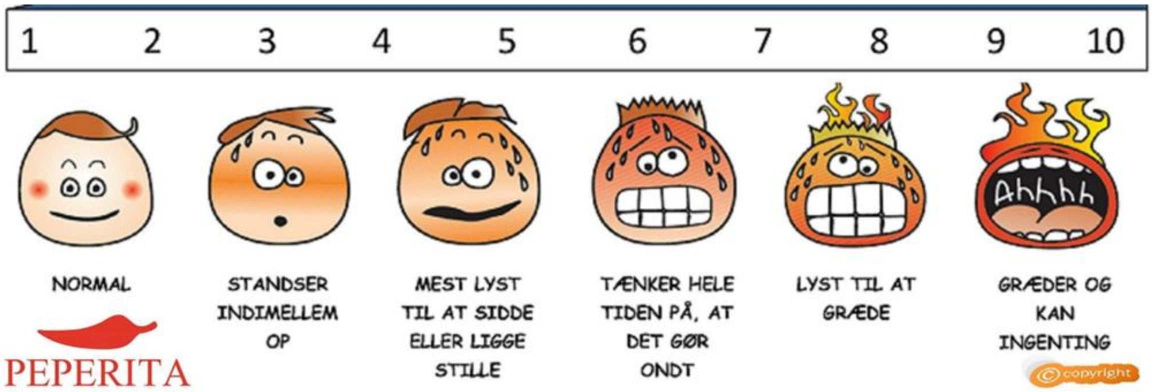
Plantartrykmåling	☐ Udført<br>☐ Ikke udført
Fodscanning	☐ Udført<br>☐ Ikke udført
Skostørrelse Europæisk nummer	
Skomål<br>Måles med instrument på højre sko i mm	Længde: ______________<br>Bredde: ______________<br>Omkredse: _____________

PATIENT ID	

Hvor mange gange gik du til sport i din fritid i <u>sidste uge</u>

Hvilken sportsgren	Antal gange:	Antal timer:
Fodbold		
Håndbold		
Basketbold		
Volleybold		
Gymnastik		
Tumling		
Svømning		
Ridning		
Dans		
Andet ______________________________		
